# Stage-Associated Cellular and Molecular Signatures in Diabetic Retinopathy Identified Through Integrated Bulk and Single-Cell Transcriptomic Analysis

**DOI:** 10.3390/ijms27062775

**Published:** 2026-03-19

**Authors:** Ying Li, Lian Liu, Yuan Zhang, Lingyi Ouyang, Xiaomin Chen, Jingqiu Huang, Min Ke

**Affiliations:** Department of Ophthalmology, Zhongnan Hospital, Wuhan University, Wuhan 430071, China; zn003579@whu.edu.cn (Y.L.);

**Keywords:** diabetic retinopathy, single-cell transcriptomics, diabetic retinopathy staging, retinal cells, ANGPTL

## Abstract

Diabetic retinopathy (DR) is one of the most common microvascular complications of diabetes and can lead to severe visual impairment. Based on disease severity, DR is classified into no clinically apparent diabetic retinopathy (NDR), non-proliferative diabetic retinopathy (NPDR), and proliferative diabetic retinopathy (PDR). Although nearly all retinal cell types are involved in DR progression, the dominant cell populations and their pathophysiological changes at each stage remain unclear. By integrating bulk and single-cell transcriptomic data from human and mouse retinas, this study revealed the following: (1) In the NDR stage, photoreceptors exhibit significant changes in ribosomal pathways. (2) In the NPDR stage, endothelial cells and pericytes show marked transcriptional alterations, accompanied by enhanced LAMININ signaling in cell-cell communication. (3) At the PDR stage, neural and glial cells are extensively involved in disease progression, with notable changes in ANGPTL signaling. Additionally, this study observed DR-specific subtypes of endothelial cells and pericytes and potentially identifies gene signatures in macroglia cells that correlate with disease duration. The altered expression of several key genes in early diabetic retina was confirmed by qPCR. These findings may offer a comprehensive view of the cellular and molecular landscape underlying DR and may suggest potential targets.

## 1. Introduction

Diabetic retinopathy (DR) is one of the most common microvascular complications of diabetes and a leading cause of vision loss in the working-age population worldwide [1]. With the rise in global living standards and lifestyle-related risk factors, the prevalence of DR continues to increase [2], and the number of affected individuals is projected to reach 160 million by 2045 [3]. Clinically, DR is classified into non-proliferative diabetic retinopathy (NPDR) and proliferative diabetic retinopathy (PDR) [1]. Progression to PDR is particularly concerning, as it is associated with irreversible visual impairment, limited therapeutic options, and, in severe cases, blindness or enucleation [4].

The pathogenesis of DR involves a complex interplay of neurovascular, inflammatory, and immune mechanisms that remain incompletely understood. Traditional models have emphasized retinal microvascular abnormalities, including vascular leakage and aberrant neovascularization. However, accumulating evidence highlights the roles of oxidative stress, dysregulated autophagy and apoptosis, and chronic inflammation [5], all of which contribute to retinal neurodegeneration and disruption of the neurovascular unit [6,7,8].

The retina is a highly specialized neurovascular tissue composed of neurons, vascular cells, and glial cells, with its homeostasis depending on coordinated cellular interactions. In DR, a broad spectrum of cellular alterations has been documented [9], such as endothelial and pericyte loss [10], glial cell activation [11,12], and retinal ganglion cell (RGC) apoptosis [12]. Although these pathological processes may occur in different cell types and vary by disease stage, a comprehensive understanding of the most vulnerable cell populations and predominant pathological events at each stage of DR progression is still lacking.

With the growing availability of high-throughput datasets, public resources now provide valuable opportunities to investigate the molecular basis of DR. Bulk RNA sequencing (RNA-seq) enables the assessment of global transcriptomic changes in diabetic retinas, whereas single-cell RNA sequencing (scRNA-seq) offers the resolution to dissect cell type-specific molecular alterations and pathway dysregulation. In this study, we first applied weighted gene co-expression network analysis (WGCNA) to identify gene modules strongly associated with early, intermediate, and advanced stages of DR. We then integrated scRNA-seq datasets to detect the key cell types expressing these stage-specific modules and characterize the pathological changes within these populations. This comprehensive, stage-associated analysis provides new insights into the dynamic cellular and molecular mechanisms underlying DR progression and reveals candidate targets for future therapeutic exploration.

## 2. Results

### 2.1. Identification of Key Gene Modules and Their Mapping to Single-Cell Transcriptomes

We selected four datasets for detailed downstream analysis, including bulk RNA-sequencing data from human retinas, as well as single-cell RNA-sequencing data from db/db mice at 13 and 21 weeks of age and STZ-induced diabetic mice at 29 weeks. Detailed information on these datasets is provided in Table 1.

**Figure 1:**
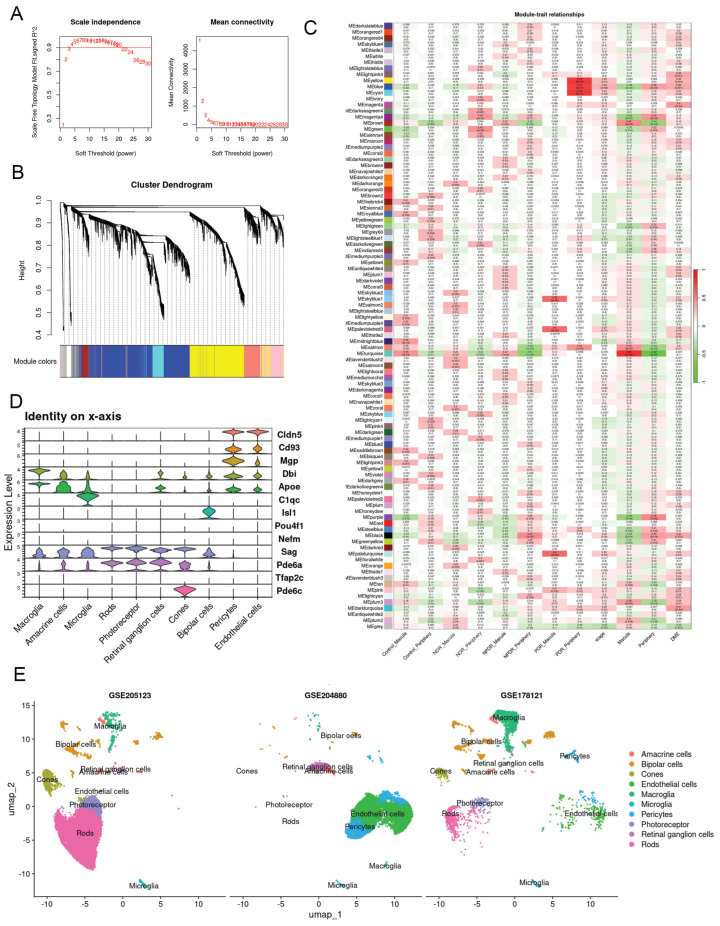
Weighted gene coexpression network analysis (WGCNA) of GSE160306. (**A**) Soft threshold selection in the WGCNA. Scale independence and mean connectivity were used for soft threshold selection in WGCNA. (**B**) Hierarchical cluster trees showing coexpression modules identified by WGCNA. (**C**) Module–trait relationships in GSE160306. Each cell contains the corresponding correlation and *p*-value. The table is color-coded by correlation according to the color legend. (**D**) Violin plot shows expression levels and distribution of representative cell markers across 10 cell populations. (**E**) UMAP plot showing the distribution of different retinal cell types.

The dataset GSE160306 contains transcriptomic profiles from both macular and peripheral retinal regions in control and diabetic patients. Subjects were classified into four groups: healthy controls, diabetics with no clinically apparent diabetic retinopathy (NDR), non-proliferative diabetic retinopathy (NPDR), and proliferative diabetic retinopathy (PDR). We performed weighted gene co-expression network analysis (WGCNA) on this dataset (Figure 1A,B). WGCNA groups co-expressed genes into distinct modules and identifies those most strongly associated with predefined clinical traits, with module genes showing significant correlation with the traits of interest.

Because mice lack a macula, we focused on peripheral retinal gene expression to enable cross-species comparison. The analysis revealed that the “NDR-periphery” group was positively correlated with the green module (correlation = 0.47, *p* = 3.69 × 10^−5^), while the “NPDR-periphery” group was negatively correlated with the turquoise module (correlation = −0.51, *p* = 1.25 × 10^−6^). The “PDR-periphery” group showed strong positive correlations with the yellow (correlation = 0.79, *p* = 5.96 × 10^−18^), cyan (correlation = 0.78, *p* = 5.16 × 10^−17^), and blue (correlation = 0.74, *p* = 5.29 × 10^−15^) modules (Figure 1A,B).

We next established an association between three murine single-cell RNA-seq datasets and the human DR stages defined in GSE160306. First, we converted the differentially expressed genes from the WGCNA modules of interest into their corresponding mouse orthologs. A total of 3806 human WGCNA genes were subjected to ortholog conversion. Among them, 3344 (87.9%) were successfully mapped to mouse orthologs. The unmapped genes were listed in Supplemental Table S3. Importantly, the majority of unmapped genes consisted of lncRNAs, snoRNAs, pseudogenes, and T cell receptor segments, which lack direct one-to-one orthologs between species.

Retinal neovascularization was confirmed in 16-week STZ-induced diabetic mice after the onset of hyperglycemia, as demonstrated by IB4 staining of retinal vascular whole mounts and CD105 immunohistochemistry [16,17]. Given that hyperglycemia lasted for 25 weeks in the GSE178121 dataset, this dataset was considered associated with the PDR stage.

Studies have shown that 10-week-old db/db mice show increased Müller cell reactivity, elevated apoptosis across retinal layers, and decreased electroretinography (ERG) amplitudes, while 13-week-old db/db mice exhibit reduced ERG responses and retinal thinning [18,19]. These studies show that the GSE205123 dataset (13-week-old mice) corresponds to the NDR group. In db/db mice at 18 weeks of age, reactive gliosis and pericyte loss have been reported [16]. Pericyte loss contributes to microaneurysms and retinal hemorrhages [20], hallmark features of early-stage DR [1]. Therefore, we defined 18 weeks as the cutoff point between the “diabetic” and NPDR groups. Consistently, the 19-21-week db/db are observed features of VEGF upregulation and enhanced BRB permeability [21]. Based on these prior findings, the GSE204880 (21-week-old mice) is relevant to NPDR.

The three single-cell RNA-seq datasets were batch-corrected and integrated into a single dataset. Cell markers and clustering results are shown in Figure 1D,E. The NDR dataset included diverse retinal cell types but relatively few endothelial cells (ECs). In contrast, the NPDR dataset was enriched for ECs and pericytes (PCs), as cells were pre-selected using PECAM1 and CD104b antibodies. In the PDR dataset, the number of rods was markedly reduced due to CD73 antibody-based rod depletion (Table 2).

### 2.2. Cell Type-Specific Transcriptomic Alterations During the NDR Stage

**Figure 2:**
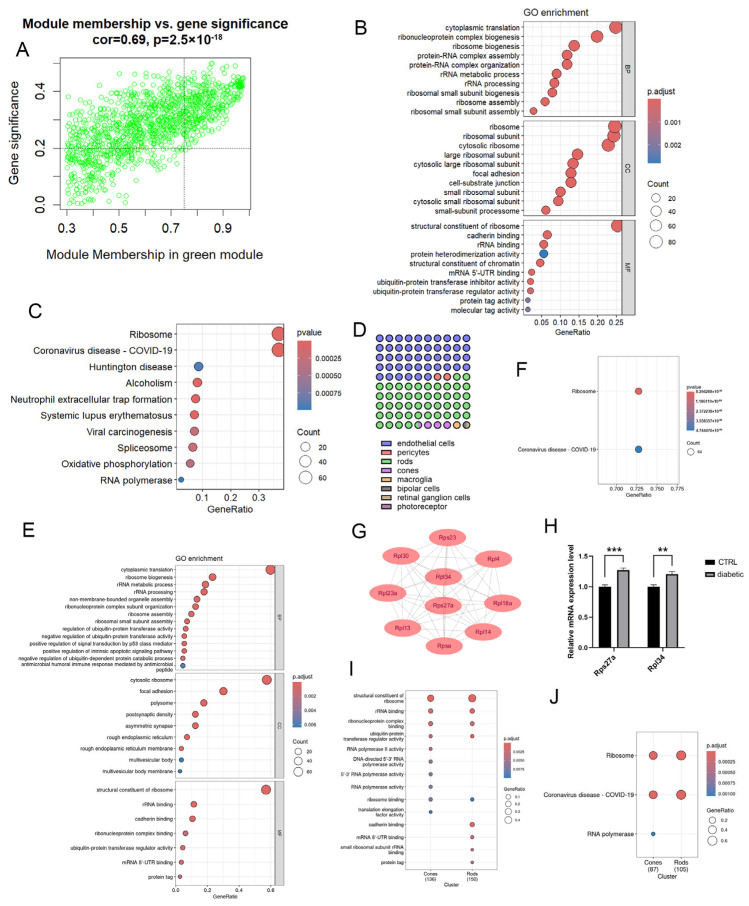
(**A**) Correlation of module membership and gene significance of genes in the green module. (**B**) GO pathway enrichment analysis of core genes in the green module. (**C**) KEGG pathway enrichment analysis of core genes in the green module. (**D**) Proportion of cell types with the top 2 highest expression of green-module genes. (**E**) GO pathway enrichment analysis of diabetic rod genes. (**F**) KEGG pathway enrichment analysis of diabetic rod genes. (**G**) Hub genes of diabetic rod genes identified by PPI analysis via Cytohubba. (**H**) Relative mRNA expression level of *Rps27a* and *Rpl34* in control and diabetic mice (** *p* < 0.01, *** *p* < 0.001, n = 6). (**I**) Comparison of GO enrichment between diabetic rod genes and diabetic cone genes. (**J**) Comparison of KEGG enrichment between diabetic rod genes and diabetic cone genes.

The no clinically apparent diabetic retinopathy (NDR) stage refers to early disease characterized by sustained hyperglycemia without apparent vascular abnormalities such as hemorrhages or microaneurysms. In WGCNA, this stage correlated significantly with the green module (Figure 2A). Core genes of the green module were defined as those with MM > 0.75 and GS > 0.2. Gene ontology (GO) enrichment analysis showed significant enrichment in cytoplasmic translation, ribonucleoprotein complex biogenesis, and ribosome biogenesis (BP); ribosome, ribosomal subunit, and cytosolic ribosome (CC); and structural constituent of ribosome, cadherin binding, and rRNA binding (MF) (Figure 2B). KEGG pathway analysis revealed enrichment in ribosome, coronavirus disease (COVID-19), and Huntington disease (Figure 2C).

Single-cell expression mapping showed that green module genes were predominantly expressed in endothelial cells, rods, and cones (Figure 2D). In the GSE205123 dataset, we identified DEGs in rods and cones and intersected them with green module genes. Overlapping sets were defined as NDR rod and cone genes, respectively (Supplementary Table S1). GO analysis of NDR rod genes revealed enrichment in cytoplasmic translation, ribosome biogenesis, and rRNA metabolism (BP); cytosolic ribosome, focal adhesion, and polysome (CC); and ribosome structural constituent, rRNA binding, and cadherin binding (MF) (Figure 2E). KEGG pathways again included ribosome and coronavirus disease (COVID-19) (Figure 2F). Protein–protein interaction (PPI) analysis identified *Rpl34* and *Rps27a* as hub genes in rods (Figure 2G). qPCR validation confirmed that both genes were upregulated in the retinas of early-stage diabetic mice (Figure 2H). Both rods and cones showed enrichment in ribosome-related pathways, but rod genes uniquely enriched cadherin binding, whereas cone genes enriched RNA polymerase II activity (Figure 2I,J).

### 2.3. Cell Type-Specific Transcriptomic Alterations During the NPDR Stage

**Figure 3:**
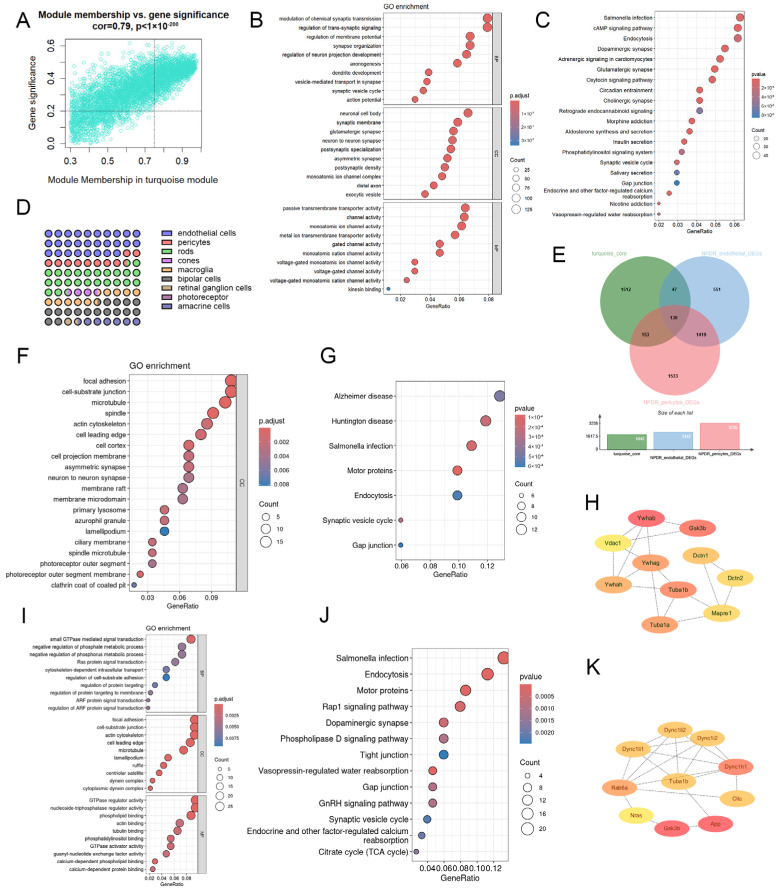
(**A**) Correlation of module membership and gene significance of genes in the turquoise module. (**B**) GO pathway enrichment analysis of core genes in the turquoise module. (**C**) KEGG pathway enrichment analysis of core genes in the turquoise module. (**D**) Proportion of cell types with the highest expression of turquoise-module genes. (**E**) Venn diagram showing the overlap among core turquoise-module genes, NPDR EC DEGs, and NPDR PC DEGs. (**F**) GO pathway enrichment analysis of NPDR EC genes. (**G**) KEGG pathway enrichment analysis of NPDR EC genes. (**H**) Hub genes of NPDR EC genes identified by PPI analysis via Cytohubba. (**I**) GO pathway enrichment analysis of NPDR PC genes. (**J**) KEGG pathway enrichment analysis of NPDR PC genes. (**K**) Hub genes of NPDR PC genes identified by PPI analysis via Cytohubba.

The NPDR stage is characterized by retinal pathology without neovascularization. NPDR was significantly associated with the turquoise module in WGCNA (Figure 3A). GO enrichment of core genes revealed significant enrichment in modulation of chemical synaptic transmission, regulation of trans-synaptic signaling, and regulation of membrane potential (BP); neuronal cell body, synaptic membrane, and glutamatergic synapse (CC); and passive transmembrane transporter activity, channel activity, and ion channel activity (MF) (Figure 3B). KEGG analysis revealed Salmonella infection, cAMP signaling, and endocytosis (Figure 3C). These genes were mainly expressed in ECs, PCs, and rods (Figure 3D).

We intersected turquoise module core genes with DEGs from ECs and PCs in the NPDR dataset (GSE204880), yielding 177 NPDR EC genes and 283 NPDR PC genes (Figure 3E). GO analysis of NPDR EC genes showed enrichment in focal adhesion, cell–substrate junction, and microtubules (CC) (Figure 3F), while KEGG showed Alzheimer’s disease, Huntington disease, and Salmonella infection (Figure 3G). PPI analysis identified Ywhag and Tuba1b as hub genes (Figure 3H).

For NPDR PC genes, enriched terms included GTPase-mediated signaling, negative regulation of phosphate metabolism, and negative regulation of phosphorus metabolism (BP); focal adhesion, cell–substrate junction, and actin cytoskeleton (CC); and GTPase regulator activity, NTPase regulator activity, and phospholipid binding (MF) (Figure 3I). KEGG pathways included Salmonella infection, endocytosis, and motor proteins (Figure 3J). Tuba1b and Dync1h1 were hub genes (Figure 3K).

**Figure 4:**
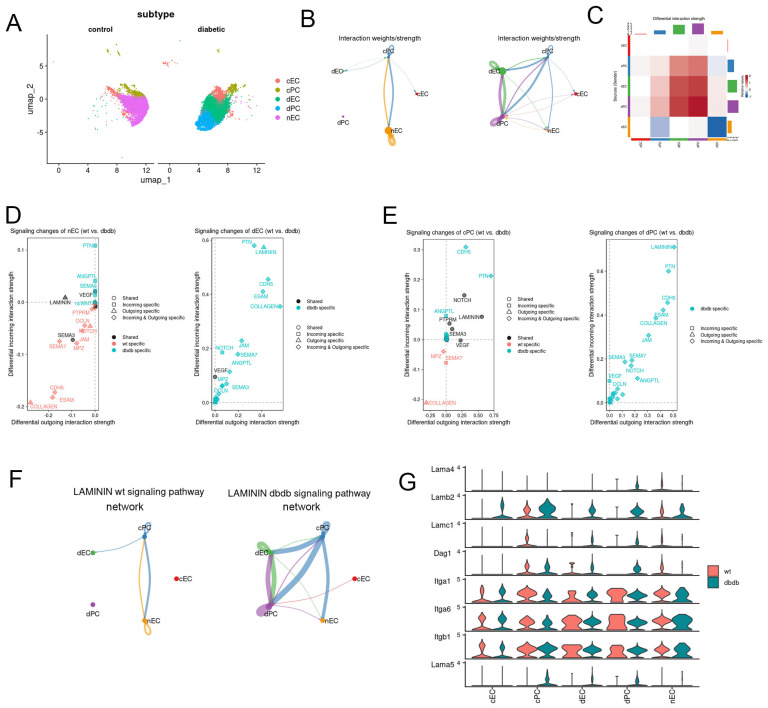
(**A**) UMAP plot showing the distribution of subtypes of EC and PC in dataset GSE204880. (**B**) Circle plot of cell communication probabilities between EC and PC subtypes in control (left panel) and diabetic (right panel) conditions. (**C**) Heatmap showing the differences in cell-cell communication probabilities between control and diabetic conditions. (**D**) Signaling changes of EC subtypes in control and diabetic conditions. (**E**) Signaling changes of PC subtypes in control and diabetic conditions. (**F**) Comparison of LAMININ signaling pathway communication strength among EC and PC subtypes in control and diabetic conditions. (**G**) Expression profiles of LAMININ signaling components in EC and PC subtypes under control and diabetic conditions.

Subtype analysis revealed three EC subpopulations: normal ECs (nEC), diabetic ECs (dEC), and common ECs (cEC). PCs were divided into diabetic PCs (dPC) and common PCs (cPC) (Figure 4A). CellChat analysis showed that under normal conditions, interactions were primarily between cPC and nEC. In diabetes, new networks emerged among cPC–dEC, dPC–dEC, and dPC–cPC, with dEC–dPC interactions being most prominent (Figure 4B).

Pathway analysis revealed upregulated CDH5, PTN, and LAMININ signaling in dEC and dPC (Figure 4C,D). Quantification of LAMININ-mediated interactions showed significantly enhanced EC–PC communication under hyperglycemia (Figure 4E). Key signaling molecules included *Lamb2*, *Lamc1*, and *Dag1* (Figure 4F).

### 2.4. Cell Type-Specific Transcriptomic Alterations During the PDR Stage

**Figure 5:**
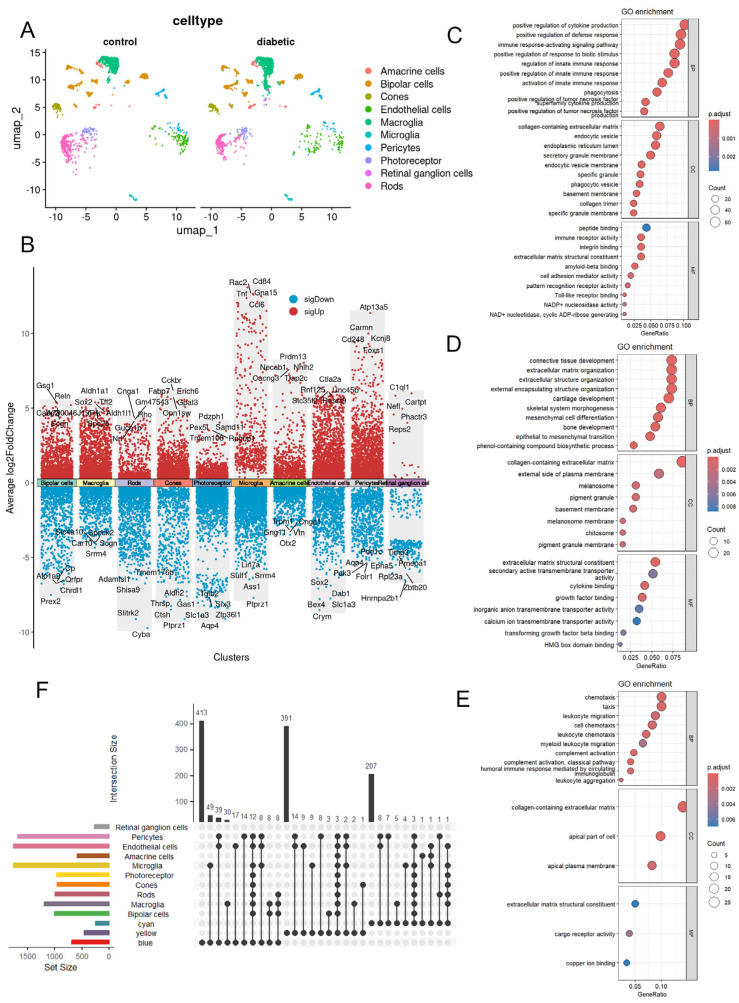
(**A**) UMAP plot showing the distribution of different retinal cell types in dataset GSE178121. (**B**) DEGs of different retinal cell types in dataset GSE178121. (**C**) GO pathway enrichment analysis of core genes in the blue module. (**D**) GO pathway enrichment analysis of core genes in the yellow module. (**E**) GO pathway enrichment analysis of core genes in the cyan module. (**F**) UpSet plot showing the intersection of cell type-specific DEGs with core genes from the blue, yellow, and cyan modules.

PDR is defined as neovascularization on the retina or optic disc, representing advanced DR [22]. Clustering analysis of the PDR single-cell dataset identified DEGs for each cluster (Figure 5A,B). WGCNA showed strong associations with yellow, cyan, and blue modules.

GO analysis of hub genes in the blue module revealed enrichment in cytokine production regulation, defense response, and immune response signaling (BP); collagen-containing extracellular matrix, endocytic vesicle, and ER lumen (CC); and peptide binding, immune receptor activity, and integrin binding (MF) (Figure 5C). The yellow module was enriched in connective tissue development, extracellular matrix organization, and extracellular structure organization (BP); collagen-containing extracellular matrix, plasma membrane external side, and melanosome (CC); and extracellular matrix structural constituent, transmembrane transporter activity, and cytokine binding (MF) (Figure 5D). The cyan module was enriched in chemotaxis, leukocyte migration, and taxis (BP); collagen-containing extracellular matrix, apical plasma membrane, and apical cell region (CC); and extracellular matrix structural constituent, cargo receptor activity, and copper ion binding (MF) (Figure 5E).

Intersecting module hub genes with PDR single-cell DEGs revealed the greatest overlap for the blue module. Microglia and EC/PC populations contributed most of the overlapping genes.

**Figure 6:**
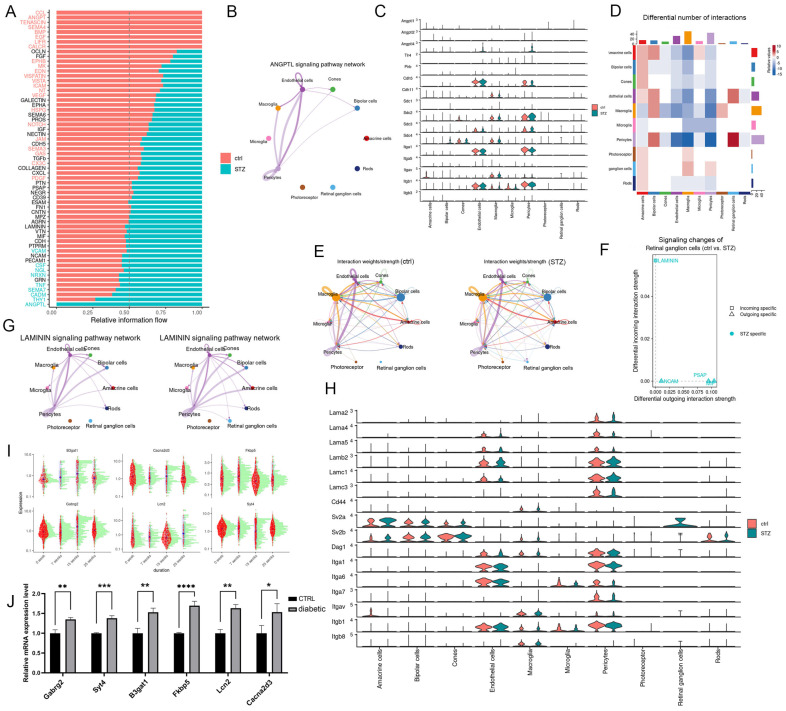
(**A**) Bar graph comparing overall signaling pathway strength between control and diabetic (STZ) conditions. (**B**) Comparison of ANGPTL signaling pathway communication strength among retinal cell types in diabetic conditions. (**C**) Expression profiles of ANGPTL signaling components in retinal cell types under control and diabetic conditions. (**D**) Heatmap of differential number of interactions among different retinal cell types. (**E**) Circle plot of cell communication probabilities between different retinal cell types in control (left panel) and diabetic (right panel) conditions. (**F**) Signaling changes of retinal ganglion cells in control and diabetic conditions. (**G**) Comparison of LAMININ signaling pathway communication strength in control (left panel) and diabetic (right panel) conditions. (**H**) Expression profiles of LAMININ signaling components under control and diabetic conditions. (**I**) Top genes in macroglia with dynamic expression changes over the duration of hyperglycemia. (**J**) Relative mRNA expression level of top genes in macroglia in control and diabetic mice (* *p* < 0.05, ** *p* < 0.01, *** *p* < 0.001, **** *p* < 0.0001, n = 6).

Pathway analysis showed ANGPTL signaling was exclusively present in diabetic mice (Figure 6A), occurring mainly between ECs and PCs and between macroglia and ECs/PCs (Figure 6B). *Angptl4* expression was markedly elevated in ECs and PCs (Figure 6C), implicating Angptl4 in EC/PC dysfunction during PDR.

Cell–cell communication quantification revealed altered interactions between RGCs and ECs/PCs in diabetic retinas (Figure 6D,E). LAMININ signaling showed the most prominent increase in incoming strength in RGCs (Figure 6F). Normally absent, LAMININ-mediated RGC–EC/PC interactions emerged under diabetes (Figure 6G). Within this pathway, *Lamb2* was upregulated in ECs/PCs, while Sv2a was expressed specifically in diabetic RGCs (Figure 6H), suggesting LAMININ signaling in mid-to-late DR pathogenesis.

### 2.5. Temporal Gene Expression Changes in Macroglia During Diabetic Retinopathy

Finally, we investigated the temporal effects of hyperglycemia on retinal cells. Because many marker genes are shared between astrocytes and Müller cells, we collectively refer to them as macroglia. Given the key role of macroglia and their consistent representation across datasets, we applied Monocle3 to track gene expression dynamics. Six genes with the most prominent temporal changes were identified: *B3gat1*, *Gabrg2*, *Cacna2d3*, *Lcn2*, *Fkbp5*, and *Syt4* (Figure 6I). qPCR confirmed that all six candidate genes were upregulated in the retinas of early-stage diabetic mice (Figure 6J). These genes are likely involved in contributing to macroglial activation during DR progression.

## 3. Discussion

### 3.1. Experimental Design: Integration of Species and Sequencing Platforms

This study combined human bulk RNA sequencing with mouse single-cell sequencing, thereby integrating both cross-species and cross-platform datasets. Single-cell sequencing has been applied to proliferative membranes obtained during surgery [23], but these samples do not represent retinal tissue itself. Since human retinal tissue is not accessible for such studies, no single-cell sequencing of the human retina has been reported to date. Thus, animal models remain indispensable for investigating cell type-specific transcriptomic changes in pre-proliferative stages of PDR. Moreover, the integration of sequencing datasets across species increases the robustness and generalizability of our conclusions.

Cross-species comparisons between human and mouse retinas inherently introduce biological and methodological limitations. Fundamental anatomical and cellular differences exist between species [24,25]. Notably, the human retina contains a specialized macula and fovea with a high cone density and distinct vascular architecture, whereas the mouse retina is rod-dominant and lacks a macular structure. These structural differences are accompanied by variations in photoreceptor distribution, metabolic demand, and microvascular organization, which may influence cellular responses to hyperglycemic stress. Nevertheless, Lu et al. demonstrated that humans and mice exhibit broadly similar retinal cellular expression patterns [26], which provides a strong rationale for integrating data across the two species. Moreover, the macula is a developmentally specialized region that is more advanced than the peripheral retina [26]. For this reason, we analyzed human peripheral retina rather than macular tissue alongside the mouse retina.

In addition, this study integrates two commonly used diabetic mouse models, db/db and STZ-induced diabetes [16], which represent distinct forms of metabolic dysregulation (genetic type 2 diabetes versus chemically induced hyperglycemia). Combined analysis was therefore intended to improve the robustness of identifying diabetes-associated transcriptional signatures. Although these models capture complementary aspects of diabetic pathology [27,28,29], neither fully recapitulates the complex and heterogeneous progression of human diabetic retinopathy. Consequently, mechanistic interpretations derived from cross-species integration remain hypothesis-generating and will require further validation in human tissues and functional experimental systems.

Bulk and single-cell sequencing each have distinct advantages and drawbacks. Bulk sequencing offers deep sequencing depth, making it more sensitive for detecting gene expression changes, but it cannot resolve which cell types contribute to those differences [30]. In contrast, single-cell sequencing has shallow depth but provides critical cell type-specific resolution. By projecting bulk sequencing results onto the single-cell transcriptomic framework, we were able to better contextualize differential gene expression and identify its cellular origins.

### 3.2. Early Transcriptional Alterations in Photoreceptors Suggest Ribosomal Response in NDR Retina

In the NDR stage of diabetes, our analysis revealed that DEGs were primarily localized to photoreceptors, with both rods and cones showing strong enrichment in ribosome-related pathways. Among these, *Rpl34* and *Rps27a*—two genes encoding ribosomal proteins—emerged as hub genes. Notably, these molecular alterations were present before the appearance of vascular pathology typically associated with diabetic retinopathy.

Ribosome response has a profound impact on the development of photoreceptors [31,32], which are among the most vulnerable retinal neurons under hyperglycemic stress [33]. Early functional impairment of the rod signaling pathway has been reported in diabetic mice, including diminished light-evoked inhibition of rod bipolar cells [34] and disrupted photoreceptor synaptic architecture [35]. Rod phototransduction has also been implicated in sensitizing retinal vasculature to diabetic damage [36]. Furthermore, early cone cell abnormalities have been observed in a zebrafish model of DR [33], while photoreceptor structural and functional alterations have been documented in patients with early diabetes [37,38]. We consider it to be associated with the exceptionally high metabolic demands of photoreceptors.

*Rpl34* and *Rps27a* are both putatively important in ribosome assembly and protein translation [39]. *Rpl34* and *Rps27a* both play critical roles in ribosome assembly and protein translation. In our study, we observed upregulation of these two genes in the retinas of early-stage diabetic mice, suggesting that our model may correspond to the NDR stage and further highlighting the potential functional relevance of these genes. However, ribosome-related transcriptional changes observed in photoreceptors under early diabetic conditions may reflect an early neuronal stress response; whether these alterations contribute to subsequent microvascular pathology remains to be determined.

### 3.3. Endothelial and Pericyte Transcriptomic Remodeling Characterizes the Intermediate Stage of Diabetic Retinopathy

In the intermediate stage of DR, marked by retinal hemorrhages and microaneurysms but the absence of neovascularization, key transcriptomic changes were concentrated in endothelial cells (ECs) and pericytes (PCs). DEGs in these cells were significantly enriched in pathways related to cell adhesion and cytoskeletal organization, pointing to extensive structural and functional remodeling.

Cell clustering revealed the emergence of diabetic-specific endothelial cell (dEC) and pericyte (dPC) subtypes, which were absent in controls, suggesting extensive transcriptional reprogramming. With the growing application of single-cell sequencing, disease-associated cellular subpopulations are increasingly being identified. For example, Xia et al. reported three distinct pericyte subtypes in the oxygen-induced proliferative retinopathy model, including an activated pericyte subset with pro-angiogenic potential, a pericyte subset with inflammatory properties, and a resting pericyte subset with homeostatic functions [40]. Similarly, Yao et al. identified a diabetes-specific endothelial subpopulation in 20-week-old db/db mice, characterized by upregulated pathways related to inflammation and cell migration [41].

These findings indicate the inherent heterogeneity of ECs and PCs, which may underlie their diverse responses to pathological stress. In our study, cells from NPDR and control groups were segregated into distinct clusters, further indicating that disease-associated changes represent global transcriptional reprogramming rather than the differential expression of a limited number of genes. This observation supports the notion that ECs and PCs play a central role in the pathophysiology of this stage of diabetic retinopathy.

### 3.4. Activation of ANGPTL and LAMININ Signaling Indicates Multicellular Remodeling and ECM Dysregulation in Proliferative Diabetic Retinopathy

In the proliferative stage of DR (PDR), characterized by pathological neovascularization, transcriptomic changes extended across multiple cell types, including glia, retinal ganglion cells (RGCs), PCs, and ECs. Extracellular matrix (ECM) remodeling appeared to be a potential mediator of progression.

Notably, ANGPTL signaling was exclusively activated under diabetic conditions and mediated interactions among ECs, PCs, macroglia, and neurons. *Angptl4* expression was markedly upregulated in ECs and PCs, consistent with previous reports implicating ANGPTL family members in DR [42,43]. ANGPTLs regulate glucose metabolism, lipid homeostasis, and angiogenesis, and ANGPTL4 in particular has been associated with vascular permeability, aberrant angiogenesis, and retinal inflammation [44,45,46,47].

LAMININ signaling emerged as a central mediator of RGC-vascular interactions in PDR, as well as of the crosstalk between dECs and dPCs in NPDR. Laminins, essential ECM glycoproteins, regulate adhesion, migration, and differentiation through integrin binding and downstream activation of FAK, PI3K/Akt, and MAPK signaling. Previous studies have implicated laminin-related proteins in DR: Netrin-4 (NTN4) contributes to vascular stabilization and inflammation regulation [48]; laminin subunit alpha-1 (LAMA1) is enriched in basement membrane thickening in PDR [49]; and AGE-modified laminins disrupt Müller cell potassium channel function, leading to cellular swelling and dysfunction [50]. Collectively, these findings suggest that LAMININ-related signaling may be involved in the pathological changes observed in middle-to-late-stage DR, particularly within neuron-glia-vascular interactions, and may therefore represent a pathway warranting further investigation in the context of potential therapeutic strategies. CellChat predictions are derived from ligand–receptor transcript co-expression, and they infer communication probabilities [51]. Moreover, transcript abundance does not necessarily reflect protein secretion, receptor engagement, or pathway activation; therefore, these predicted LAMININ- and ANGPTL-related interactions warrant further validation at the protein and functional levels.

### 3.5. The Critical Role of Macroglia Cells in Diabetic Retinopathy and Their Associated Gene Signatures

Macroglia, namely, astrocytes and Müller cells, play a central role in DR pathogenesis, contributing to macular edema, neovascularization, and fibrotic proliferation [52]. We identified six genes with dynamic expression changes in macroglia under hyperglycemic stress: *Lcn2*, *Syt4*, *B3gat1*, *Cacna2d3*, *Gabrg2*, and *Fkbp5*. Moreover, the upregulation of all these genes was confirmed in early-stage diabetic mice. Among them, *Lcn2* and *Syt4* have established links to DR. *Lcn2*, a marker of reactive gliosis, is upregulated in macroglia of diabetic Akimba mice [53]. *Syt4*, also upregulated in diabetic retina, regulates calcium influx in high-glucose-treated ARPE-19 cells, promoting GLUT1 membrane translocation, enhancing glucose uptake, triggering apoptosis, and accelerating DR progression [54].

The remaining four genes may represent novel contributors. *B3gat1* (also known as CD57) is implicated in neuropathies [55] and associated with synaptic plasticity and dendritic spine morphology [56]. *Cacna2d3* encodes the α2δ3 subunit of voltage-gated calcium channels, regulating calcium influx and neurotransmitter release [57]. *Gabrg2* encodes a GABA-A receptor subunit [58] and may influence glia–neuron communication. *Fkbp5*, a stress-responsive immunophilin, modulates endocrine stress signaling and is increasingly recognized as a target in metabolic diseases, including type 2 diabetes; its inhibition improves pancreatic β-cell survival under inflammatory stress [59].

Together, these findings suggest a potential involvement of macroglia in DR and uncover candidate genes associated with glial reactivity and neurovascular alterations in diabetes. These observations may provide a framework for future studies aimed at evaluating whether targeting such pathways may influence glial responses and neurovascular function.

### 3.6. Limitations

This study has several limitations. First, although overall cellular expression patterns are broadly conserved between human and mouse retinas, cones show notable interspecies divergence [26], and, thus, the NDR-stage cone-specific genes and pathways identified here require further experimental validation. Second, the three integrated mouse single-cell RNA-seq datasets differed in cellular composition due to pre-selection of certain cell types, which may influence the cell type proportion calculation from key genes of WGCNA modules. Finally, the classification of diabetic stages in mice was based on literature reports, and the original datasets did not fully document corresponding phenotypes, necessitating additional validation experiments.

## 4. Materials and Methods

### 4.1. Access to GEO Datasets

We searched the GEO database (http://www.ncbi.nlm.nih.gov/geo, accessed on 1 February 2025) using the keyword “diabetic retinopathy” to retrieve relevant gene expression datasets. Studies were required to use retinal tissue samples, with no restriction on the species of origin. Exclusion criteria included studies based on cultured cells, oxygen-induced retinopathy models, array-based platforms, microRNA sequencing, and epigenetic sequencing. Both single-cell RNA sequencing (scRNA-seq) and bulk RNA sequencing (bulk RNA-seq) datasets were included. Four datasets—GSE160306 [13], GSE205123, GSE204880 [14], and GSE178121 [15]—were ultimately selected for analysis. Raw data were downloaded from GEO for subsequent analysis.

### 4.2. Animals and Ethical Statement

Experiments utilized male C57BL/6J (6–8 weeks) mice obtained from Shouzheng Pharma Biotechnology Co., Ltd. (Wuhan, China). Mice were housed under a 12 h light/dark cycle and controlled environmental conditions. This study was approved by the Institutional Animal Care and Use Committee (IACUC) of Shouzheng Pharma Biotechnology Co., Ltd. (SZHY no. 2025010102). All procedures were conducted in accordance with relevant guidelines to minimize animal suffering.

### 4.3. Animal Model of Diabetic Retinopathy

Mice were rendered diabetic using a combined protocol of a high-fat diet (8 weeks) and injection of STZ (50 mg/kg i.p. for 5 days) [60,61]. A diabetic state (blood glucose ≥ 16.7 mmol/L) was validated after one week, with additional STZ administered if necessary. Diabetic mice were maintained on a high-fat diet and monitored for body weight and blood glucose. After one month, the mice were euthanized, and retinas were collected for RNA extraction and qPCR analysis.

### 4.4. Quantitative Real-Time PCR

Total RNA was extracted (RNAsimple Kit, Tiangen, Beijing, China) and quantified (NanoDrop 2000, Thermo Fisher, Waltham, MA, USA). cDNA was synthesized from 1 μg RNA (HiScript II Kit, Vazyme, Nanjing, China) and used as a template for qPCR with the ReverTra Ace Kit (TOYOBO, Osaka, Japan) on a CFX96 system (Bio-Rad, Hercules, CA, USA). Gene expression levels were quantified relative to Gapdh, which served as the internal control. Relative mRNA abundance was determined using the 2^−ΔΔCt^ method. Primer sequences used for qRT-PCR are provided in Supplementary Table S2.

### 4.5. Weighted Gene Co-Expression Network Analysis

Weighted gene co-expression network analysis (WGCNA) was performed to identify gene modules with coordinated expression patterns and investigate their associations with clinical phenotypes. The WGCNA R package was used to construct diabetic retinopathy-related gene networks, incorporating all genes for the analysis. A soft-thresholding power (β) of 4 was selected. The network was constructed using the following parameters: maxBlockSize = 6000, networkType = “unsigned”, minModuleSize = 30, reassignThreshold = 0, and mergeCutHeight = 0.25. The module eigengene (ME) was used to represent the overall expression profile of each module. Module membership (MM) quantified the correlation between individual genes and their respective modules, while gene significance (GS) reflected the association between gene expression and diabetic retinopathy phenotypes. Core genes were defined as those with MM > 0.75 and GS > 0.2. For cross-species analysis, we performed orthologous gene conversion, in which human genes were mapped to their mouse counterparts using the orthologs() function from the babelgene package (version 22.9).

### 4.6. Single-Cell RNA Sequencing Analysis

Initial quality control was performed by retaining genes expressed in at least three cells (min.cells = 3) and cells with at least 200 detected genes (min.features = 200). Cells exhibiting a mitochondrial gene expression percentage exceeding 8% were excluded. The dataset was then normalized using the NormalizeData function, followed by identification of highly variable genes using FindVariableFeatures, data scaling with ScaleData, and dimensionality reduction via RunPCA. To correct for batch effects across datasets, reciprocal PCA (RPCA) integration was applied. Subsequent clustering was performed using the FindClusters() function with a resolution of 1.6, and two-dimensional visualization was achieved with RunUMAP(). Marker genes for each cluster were identified using the FindAllMarkers() and FindConservedMarkers() functions. Each cell type was manually annotated based on marker genes and established reference gene signatures.

### 4.7. Subcluster Analysis

Following cell clustering, the GSE204880 dataset primarily comprised clusters 4, 5, 6, 7, 12, 18, 39, 8, 17, and 26. Based on marker gene expression, clusters 4, 5, 6, 7, 12, 18, and 39 were classified as endothelial cells (ECs), while clusters 8, 17, and 26 were classified as pericytes (PCs). We then compared their distribution between the control and diabetic groups. Subclusters in which more than 95% of cells originated from the control group were designated as normal ECs/PCs, whereas those with more than 95% of cells from the diabetic group were designated as diabetic ECs/PCs. The remaining subclusters were defined as common ECs/PCs.

### 4.8. Differential Analysis

Differentially expressed genes were identified using the FindMarkers() function. For rods and cones in the NDR group, which exhibited relatively small differences, the parameters were set to logfc.threshold = 0.1 and min.pct = 0.2. In contrast, for diabetic and normal cell subpopulations in the NPDR group, which showed more pronounced differences, the parameters were set to logfc.threshold = 0.25 and min.pct = 0.2.

### 4.9. Identification of Cell Types Enriched for WGCNA Modules

We defined a custom function get_proportion() to quantify the distribution of module genes across cell types. For each input gene, its expression matrix was binarized by setting values >0 as “expressed” and those = 0 as “not expressed.” The function then calculated two levels of proportions: (i) the overall proportion of cells expressing the gene across the entire dataset and (ii) the proportion of expressing cells within each annotated cell type. Based on these cell type-specific proportions, the function ranked all cell types and identified the top three with the highest proportions. When applying this function to the key genes of each module, we found that the third-ranked cell type usually accounted for a very small proportion compared with the top two. Therefore, we focused on the top two enriched cell types and visualized their proportions across modules.

### 4.10. Pathway Analysis

Gene ontology (GO) and Kyoto Encyclopedia of Genes and Genomes (KEGG) pathway enrichment analyses were conducted on the identified differentially expressed genes using the clusterProfiler R package (version 4.8.3) [62]. *p*-values were adjusted for multiple testing using the Benjamini–Hochberg false discovery rate (FDR) correction.

### 4.11. Protein–Protein Interaction (PPI) Network Construction and Analysis

Core genes were subjected to functional protein association network analysis using the STRING database. To identify hub genes, we utilized the CytoNCA plugin in Cytoscape (version 3.9.1), selecting genes based on betweenness centrality. In addition, CytoHubba, another Cytoscape plugin, was employed to further screen hub genes using the Maximal Clique Centrality (MCC) algorithm.

### 4.12. CellChat Analysis

To investigate intercellular communication from scRNA-seq data, we performed CellChat analysis. A new CellChat object was initialized using the Seurat object, with cell type annotations incorporated as metadata. CellChat identified differentially overexpressed ligand–receptor pairs for each cell population and assigned a probability score to quantify communication strength between cell groups. Significant interactions were determined via a permutation-based statistical test with the computeCommunProb() function (number of permutations for *p*-value calculation is 100), in which cell group labels were randomly shuffled and interaction probabilities recalculated. Visualization of communication networks was achieved using circle plots and heatmaps, generated by the netVisual_circle() and netVisual_heatmap() functions, respectively. To characterize signaling patterns, we employed the netAnalysis_signalingRole_heatmap() function to analyze both outgoing and incoming interactions across cell types. Finally, pathway-specific interactions were visualized using the netVisual_aggregate() function.

### 4.13. Analysis of the Impact of Hyperglycemia Duration on Macroglia Gene Expression

To investigate how the duration of hyperglycemia affects gene expression patterns in macroglia, we performed a regression analysis using the monocle3 R package. Macroglia were extracted from the Seurat object to construct a monocle3 object (cds). We applied the fit_models() function to fit a regression model, specifying the duration of hyperglycemia as the independent variable through the model_formula_str parameter. Genes with the most significant temporal expression changes were identified using the coefficient_table() function. Finally, gene expression dynamics over time were visualized using the plot_genes_hybrid() function.

### 4.14. Software and Data Analysis Tools

Single-cell and WGCNA analyses were conducted using R (version 4.3.1). Venn diagrams were generated using the jvenn web-based tool [63], and UpSet plots were created with the ggplot2 package (version 3.5.1). For subclustering of ECs and PCs, the cluster_cells() function from the R package Monocle3 was applied. Differentially expressed genes were visualized using the jjVolcano() function from the scRNAtoolVis (version 0.1.0) R package.

All qPCR data were presented as the mean ± standard deviation (SD) of at least three independent experiments. Statistical significance between two groups was determined using a two-tailed unpaired Student’s *t*-test. For *p*-value calculation, multiple testing correction was conducted. A *p*-value < 0.05 was considered statistically significant (* *p* < 0.05, ** *p* < 0.01, *** *p* < 0.001, **** *p* < 0.0001). Analyses were performed using GraphPad Prism v9.5.1 (GraphPad Software, La Jolla, CA, USA).

## 5. Conclusions

In summary, this integrative bulk and single-cell transcriptomic analysis delineates stage-associated cellular hierarchies and signaling alterations during diabetic retinopathy progression. A ribosome-associated response in photoreceptors appears to be detectable in the early diabetic retina, whereas vascular cell remodeling accompanied by increased laminin-related signaling may become more prominent during NPDR. In the PDR stage, broader neural–glial alterations together with changes in ANGPTL-related signaling are observed. The identification of endothelial and pericyte subpopulations showing stage-associated transcriptional patterns, along with macroglial gene signatures potentially linked to disease duration, suggests dynamic and coordinated cellular responses during DR progression. Collectively, these observations may help extend the current understanding of DR pathogenesis and provide a potential framework for future studies exploring stage-targeted therapeutic strategies.

## Figures and Tables

**Table 1:** Datasets used in this study.

GEO Accession	Species	Sequencing Platforms	Conditions/Treatments	Duration of Hyperglycemia	Cell Numbers	References
GSE160306	Homo sapiens	bulk sequencing	ctrl/NDR/NPDR/PDR	-	-	[13]
GSE205123	Mus musculus	single-cell sequencing	db/db	13-6 weeks	18,170	none
GSE204880	Mus musculus	single-cell sequencing	db/db	21-6 weeks	15,210	[14]
GSE178121	Mus musculus	single-cell sequencing	STZ	25 weeks	6359	[15]

**Table 2:** The cell/tissue composition of datasets used in this study.

GEO Accession	Antibody-Based Sorting	Cell/Tissue Type Composition
GSE160306	none	macula and retinal periphery
GSE205123	none	a diverse composition of retinal cell types
GSE204880	PECAM1 and CD104b to enrich EC and PC	ECs and PCs of glomerular and retinal
GSE178121	CD73 to reduce rods	a diverse composition of retinal cell types with low proportion of rods

## Data Availability

Availability of Data and Materials: The GSE160306, GSE205123, GSE204880, and GSE178121 datasets are available in the GEO repository (https://www.ncbi.nlm.nih.gov/geo/, accessed on 1 February 2025).

## Supplementary Materials

The following supporting information can be downloaded at https://www.mdpi.com/article/10.3390/ijms27062775/s1.

## Abbreviations

The following abbreviations are used in this manuscript:

DR	diabetic retinopathy
NDR	no clinically apparent diabetic retinopathy
NPDR	non-proliferative diabetic retinopathy
PDR	proliferative diabetic retinopathy
RGC	retinal ganglion cell
scRNA-seq	single-cell RNA sequencing
WGCNA	weighted gene co-expression network analysis
bulk RNA-seq	bulk RNA sequencing
ME	module eigengene
MM	module membership
GS	gene significance
RPCA	reciprocal PCA
ECs	endothelial cells
PCs	pericytes
GO	gene ontology
KEGG	Kyoto Encyclopedia of Genes and Genomes
FDR	false discovery rate
MCC	maximal clique centrality
SD	standard deviation
BP	biological process
CC	cellular component
MF	molecular function
PPI	protein–protein interaction
nECs	normal ECs
dECs	diabetic ECs
cECs	common ECs
dPCs	divided into diabetic PCs
cPCs	common PCs
ECM	extracellular matrix
NTN4	netrin-4
LAMA1	laminin subunit alpha-1
