# Supplementary material for: Stage-Associated Cellular and Molecular Signatures in Diabetic Retinopathy Identified Through Integrated Bulk and Single-Cell Transcriptomic Analysis
Source: Int J Mol Sci. 2026 Mar 19;27(6):2775. doi: 10.3390/ijms27062775 (PMC13026524; doi:10.3390/ijms27062775)
Supplement: Supplementary file 1 [file ijms-27-02775-s001.zip › Supplementary Table S1.pdf]

NDR rod genes	NDR cone genes	NPDR EC genes	NPDR PC genes
Rhbdd2	Polr2j	Ap2b1	Ica1
Ss18l2	Rps20	Cx3cl1	Msl3
Rps20	Vps13d	Rala	Mycbp2
Rpl18	Mrpl43	Prom1	Ap2b1
Ybx1	Rpl18	Ids	Cx3cl1
Ncbp3	Ybx1	Mdh1	Prom1
Rps5	Clns1a	Arap2	Cyth3
Chmp5	Rps5	Map4	Ids
Smarcb1	Chmp5	Eif2ak2	Hivep2
Rpl3	Tnrc6a	Ccdc85a	Tmsb10
Elob	Tecr	Ybx3	Timp2
Rps16	Smarcb1	Btbd1	Prex2
Rps19	Dnal4	St6galnac2	Arap2
Rpl18a	Phf5a	Map4k4	Map4
Tmem147	Prmt5	Lims2	Mcur1
Chchd2	Polr2c	Ube2d1	Mapk9
Sec61b	Elob	Fry	Foxn3
Edf1	Eif3e	Mgll	Syne2
Rpl28	Eef1d	Kifap3	Eif2ak2
Rpl19	Rps16	Rexo2	Ccdc85a
Rpl34	Polr2i	Nebl	Ybx3
Rps13	Nop53	Epb41l2	Ptpru
Rps12	Rpl18a	Gsk3b	St3gal6
Selenok	Chchd2	Blvrb	Btbd1
Rpl24	Sec61b	Hnrnpc	Reep1
Rps15	Rpl28	Dpysl2	Rasgrp2
Tmem59	Rpl19	Palm	Nucb2
Park7	Fbxl20	Cyb5r3	St6galnac2
Tmem9	Rangrf	Rbfox2	Map4k4
Rps25	Ndufc1	Cds2	Ube2d1
Pfdn5	Copz1	Mapre1	Ppp2r3a
Ndufa1	Mrpl51	Jag1	St6gal1
Mif4gd	Rps12	Vapa	Nuak1
Alkbh7	Rnf7	Pls3	Bcap29
Rpl23	Tmem59	Nomo1	Rexo2
Snrpd2	Tmem9	Stmn2	Dync1i2
Ergic3	Sdhb	Nova2	Nebl
Romo1	Prdx1	Ogdh	Epb41l2
Eapp	Rps25	Tspan13	Nfe2l1
Dad1	Rpn2	Slc1a1	Gsk3b
Rpl36	Rpl21	Spock2	Efr3b
Chmp2a	Pfdn5	Ccser2	Ddhd2
Rpl27	Ndufa1	Git1	Gnas
Tpt1	Alkbh7	Smurf2	Blvrb
Lamtor5	Clpp	Dusp3	Pcbp4
Rps15a	Rpl23	Coro1c	Hnrnpc
Rpl35	Snrpb	Prph2	Dpysl2
Rps6	Ergic3	Lama4	Fkbp5
Rplp1	Sbds	St8sia4	Palm
Atraid	Eapp	Gnat1	Cyb5r3
Rps24	Apoe	Atp6v1a	Rbfox2

Rps2	Rpl36	Actr3	Kctd17
Rps11	Ndufa2	Rtn4	Cds2
Rpl13a	Arfip2	Chmp3	Mapre1
Rpl11	Tpt1	Prkd3	Jag1
Rps8	Lamtor5	Epha4	Usp14
Rps27a	Rps15a	Wls	Vapa
Rpl32	Tmem258	Kdm5b	Pls3
Rpl10	Stt3a	Klf7	Cd99l2
Rpl7	Cdk4	Anxa11	Dgkh
Eif3h	Tspan31	Cisd1	Nomo1
Rpl7a	Rpl35	Tuba1b	Coro2b
Rps3	Hint2	Rreb1	Stmn2
Fau	Rplp1	Ppp1r12c	Nova2
Cwc15	Gtf2b	Capns1	Pld3
Rpl30	Esd	Thra	Hnrnpul1
Eef1a1	Vps53	Ywhah	Ogdh
Rpl8	Rps11	Cdkn2d	Tspan13
Rpl26	Rpl11	Reep5	Slc1a1
Rpl29	Rps8	Serinc3	Spock2
Rps14	Snapin	Myh10	Ccser2
Rpl36al	Sccpdh	Dstyk	Git1
Rpl27a	Rpl10	Stard13	Smurf2
Ppib	Rpl7	Pdzd2	Dusp3
Gpx4	Rps3	Tmtc1	Prkar1a
Rpl13	Gtf3c6	Swap70	Unc119
Fth1	Eef1a1	Arl8b	Usp46
Rpsa	Rpl8	Lmo2	Ube4a
Setd5	Scnm1	Dync1li2	Coro1c
Rnf181	Vps72	Tbc1d4	Cmas
Rps9	Rpl36al	Tubb2a	Rab35
Rps21	Rpl13	Prcp	Cep85l
Rps7	Polr2g	Ggh	Kctd20
Bsg	Rpsa	Arhgap20	Phactr1
Rpl38	Pkig	Hadhb	Heca
Trmt112	Rnf181	Anxa7	Prph2
Uqcrh	Triap1	Slc39a8	Lama4
Eif1	Rps9	Mapk8ip3	Rad50
Rpl4	Tsen34	Prickle1	Actr1b
Rpl15	Rps7	Llph	Actr3
Taldo1	Hectd4	Slc7a1	Tanc1
Rplp2	Rpl4	Esyt1	Prkd3
Rps27	Ctdnep1	Lysmd2	Epas1
Rps17	Zfas1	Mfge8	Pla2g4a
Selenof	Rplp2	Map1lc3b	Wls
Mrpl54	Polr2l	Cltc	Map7d1
Rps23	Dpm3	Csnk1d	Kdm5b
Pclo	Ssr4	App	Camsap2
Rpl14	Snrpe	Sh3bgrl3	Klf7
Naca	Rps17	Mgst3	Itgb1bp1
Tomm7	Mrpl54	Snx27	Dnajc15
Rpl37a	Tssc4	Plk2	Anxa11
Rpl12	Nsmce3	Pam	Spats2

Rpl23a	Ubald2	Mfhas1	Tuba1b
Ubl5	Mrpl40	Fam171a1	Atpaf1
Rpl10a	Krt10	Rom1	Ndufaf4
Rpl39	Pclo	Lats2	Acsl3
Rack1	Sumo2	Crim1	Rab22a
Psenen	Rpl12	Dst	Mcf2l
Rps29	Rpl23a	Rasgrp3	Capns1
Uba52	Mrps18b	Asap1	Thra
Rps18	Rack1	Ddah1	Ywhah
Tma7	Psenen	Tbrg1	Podxl
Rps28	Uba52	Sorbs2	Cdkn2d
Nme1	Ost4	Ankrd40	Reep5
	Rps28	Larp1	Snx9
	Nme1	Nptn	Mpp1
	Malat1	Eif4a2	Mgat1
	Brk1	Lrp8	Acly
	Rnf115	Rusc1	Grsf1
	Zfp62	Cmpk1	Syt11
		Suclg1	Serinc3
		Gmps	Myh10
		Smim12	Mprip
		Rho	Dstyk
		Stard4	Stard13
		Ebf1	Rtn3
		Dlc1	Tmtc1
		Dynlt3	Swap70
		Marf1	Arl8b
		Ywhab	Napg
		Islr2	Lpin1
		Tuba1a	Smpd2
		Nxn	Rab11fip5
		Otub1	Dysf
		Abca3	Dync1li2
		Adrb2	Serpine2
		Sin3a	Tbc1d4
		Luzp1	Sucla2
		Ugp2	Tubb2a
		Ywhag	Flot1
		Trappc1	Prcp
		Gsta4	Ggh
		Lrrc8c	Hadhb
		Synpo2	Khk
		Stox2	Anxa7
		Psmd1	Sema7a
		Gpr160	Slc39a8
		Rsrc1	Mapk8ip3
		Ctbp2	Git2
		Dctn2	Slc7a1
		Cltb	Esyt1
		Rab6a	Wdr20
		Kbtbd11	Tgfb1i1
		Irx3	Zfhx3

Cd151
Myadm
Kctd2
Anxa2
Gjc1
Tob2
Dennd5a
Ube2l3
Chp1
Slc39a10
Pcbp2
Psmd12
Pdgfa
Myo1c
Dnm3
Pim3
Inpp5f
Srebf2
Dctn1
Nras
Vdac1
Shank3

Cltc
Cacna1a
App
Sh3bgrl3
Mgst3
Xpr1
Snx27
Smyd2
Cdc42bpa
Arl8a
Retreg2
Dync1li1
Lpp
Tcta
Plk2
Pam
Arhgap26
Nacc2
Pard3
Add3
Endod1
Rom1
Lats2
Crim1
Magi1
Vti1a
Mfsd6
Dst
Rasgrp3
Utrn
Asap1
Ddah1
Tbrg1
Sorbs2
Ankrd40
Atp6v1c1
Psd3
Zfyve9
Abr
Ralgds
Ano10
Rusc1
Camk2n1
Suclg1
Gmps
Rho
Arhgef3
Stard4
Ebf1
Ccdc127
Dlc1
Tmem65

Dynlt3
Pgm2l1
Marf1
Stat6
Ywhab
Islr2
Tuba1a
Sema6b
Spryd3
Abca3
Adrb2
Arl13b
Ugp2
Chd3
Ywhag
Trappc1
Gsta4
Lrrc8c
Tada2b
Grk2
Glrx
Smarcc1
Psmd1
Gpr160
Pitpna
Atp2a2
Mfsd4a
Rsrc1
Dctn2
Ppp2r2d
Rab6a
Kcmf1
Kbtbd11
Flii
Plec
Galnt11
Myadm
Znrf2
Kctd2
Plcb1
Anxa2
Gjc1
Spns2
Gas6
Dennd5a
Pde4b
Ube2l3
Syn3
Insig1
Chp1
Ptma
Mapk12

Slc38a3
Anxa4
Dync1h1
Pcbp2
Psmd12
Tbc1d9b
Pdgfa
Myo1c
Dnm3
Pim3
Tcaf1
Calm1
Inpp5f
Opa1
Srebf2
Tbkbp1
Gdi1
Sacm1l
Nras
Tmem242
Pcp4l1
Dynll2
Srgap2
Ncoa4
