# Supplementary material for: Stage-Associated Cellular and Molecular Signatures in Diabetic Retinopathy Identified Through Integrated Bulk and Single-Cell Transcriptomic Analysis
Source: Int J Mol Sci. 2026 Mar 19;27(6):2775. doi: 10.3390/ijms27062775 (PMC13026524; doi:10.3390/ijms27062775)
Supplement: Supplementary file 1 [file ijms-27-02775-s001.zip › Supplementary Table S2.pdf]

Supplementary Table S2. Primer sequences used in qPCR

Primer name	Forward	Reverse
mouse Rps27a	GCCAAGATCCAGGATAAGGAAGG	CCGAAGTCTCAACACCAGATGAA
mouse Rpl34	GCGGGCTTTCCTTATTGAG	TTCTGACTCTGTGCTTGTG
mouse Gabrg2	CTATGTGGTGATGTCTGTGTA	AATGTAAGTCTGGATGGTGAA
mouse Syt4	TTTCCAAGTATCCCACAGA	CCACCAAACATCCTATAACC
mouse B3gat1	TTCCTGAGCCGATTCTGT	CTTGACCTTGAGCCCATTC
mouse Fkbp5	GAGATGTGGTGTTCGTTGTTG	CAATCGGAATGTCGTGGTCTT
mouse Lcn2	CCAGGACTCAACTCAGAACTT	GGACAGTGAGCAGAGATGG
mouse Cacna2d3	CTACTACTATACAGACATCAA	CCTTCTTCAATGGTTACA
mouse Gapdh	AGGTCGGTGTGAACGGATTTG	TGTAGACCATGTAGTTGAGGTCA
