# Supplementary material for: Stage-Associated Cellular and Molecular Signatures in Diabetic Retinopathy Identified Through Integrated Bulk and Single-Cell Transcriptomic Analysis
Source: Int J Mol Sci. 2026 Mar 19;27(6):2775. doi: 10.3390/ijms27062775 (PMC13026524; doi:10.3390/ijms27062775)
Supplement: Supplementary file 1 [file ijms-27-02775-s001.zip › Supplementary Table S3.pdf]

| | | | | |
|---|---|---|---|---|
| RPL13AP25 | TRAJ36 | NRIP3-DT | RPL32P1 | SIGLEC16 |
| RPL7P9 | TRAJ37 | RBPMS-AS1 | HSPA7 | SMIM43 |
| ADIRF | NHERF2 | LINC02389 | TRIM53AP | ERAP2 |
| SNHG29 | CLTCL1 | LOC101928[illegible] | RPL12P10 | DEFB104A |
| ZFAS1 | STS | ZNF432 | TEX41 | GIMAP3P |
| RPL10P16 | C10orf95 | TROAP-AS1 | MTND6P11 | SLC47A2 |
| TP53TG1 | PLEKHA8P1 | LOC124907[illegible] | LINC01985 | LOC340268 |
| RNU5A-1 | RNF175 | LOC119746[illegible] | PCGEM1 | RNU1-105P |
| SNORA73B | ZNF215 | LOC103344[illegible] | MTCYBP7 | RNA5SP74 |
| SNORA5C | GLYATL2 | LASP1NB | ITPKB-IT1 | RNU6-151P |
| SNHG32 | GLYATL1 | FMNL1-DT | LINC00710 | LINC02898 |
| RNVU1-7 | TOX-DT | ST8SIA5-D[illegible] | LINC01739 | RNU6-338P |
| SNORA54 | GSTA4 | LOC780529 | EMX2OS | LOC124900267 |
| SNORA14B | DCDC1 | LOC105377[illegible] | DOCK9-AS1 | RNU6-1104P |
| SNORA20 | RAX2 | SNORD114-[illegible] | LINC02028 | RNU6-780P |
| SNORD17 | KCNJ5-AS1 | GAS6-DT | FTLP18 | RNU6-1300P |
| SNORA53 | MIR1-1HG | DGCR5 | LINC00276 | LINC00671 |
| SNORA12 | C12orf76 | PCCA-DT | LINC02884 | PELATON |
| RPLP0P6 | SLC66A1L | TRAC | RPL26P24 | RPL23AP14 |
| RNU6ATAC | OVOS2 | PWAR5 | CYP1B1-AS1 | LINC01375 |
| RPS27AP16 | LINC00652 | ARSD | LINC00892 | UBE2V2P1 |
| RPL41 | ZNF875 | CHI3L2 | UICLM | NPM1P30 |
| RPL3P4 | FAM87A | APOBEC3F | LINC00462 | EHBP1-AS1 |
| GAS5 | NCR3LG1 | TRIM22 | LINC01761 | LINC01307 |
| RPL23AP42 | COLCA1 | PLAAT4 | AKAP8P1 | LOC102724511 |
| SMIM27 | SRGAP2B | FGFBP2 | LINC01239 | LINC01271 |
| RPL13AP5 | NBEAP2 | HERC5 | LINC00330 | KRT18P62 |
| PITPNA-AS1 | SNORD114-1 | VENTX | MXRA5Y | LINC00460 |
| SCARNA7 | SNORD114-[illegible] | MIR4435-2[illegible] | HAND2-AS1 | RPL7P36 |
| LINC00847 | SNORD114-[illegible] | LINC00943 | CDK6-AS1 | TLR12P |
| MALAT1 | LINC01257 | SULT1C4 | RNU7-138P | LOC100128334 |
| SCARNA6 | MIR1915HG | LINC01270 | ADAMTS9-A[illegible] | GPC5-AS1 |
| SCARNA5 | MIR149 | LINC02982 | ADAMTS9-A[illegible] | LINC01010 |
| SCARNA21 | MIR138-1 | CYTOR | RN7SL68P | MGC27382 |
| CLN8-AS1 | GNRHR2 | SLC44A3-A[illegible] | PRDX5P1 | TM4SF1-AS1 |
| GIHCG | MIR770 | PROSER2-A[illegible] | RPL23AP66 | KCNAB1-AS2 |
| SNHG19 | TRAJ49 | LINC02607 | RPSAP36 | RN7SL132P |
| LINC01003 | SNORD113-[illegible] | VIM-AS1 | RPL17P22 | RPL7AP56 |
| SNHG8 | MEG3 | EPCAM-DT | RN7SL314P | RPL34P12 |
| LOC100419[illegible] | ZBED1 | MIR7515HG | RPS3AP31 | LOC400541 |
| YTHDF3-DT | LOC101927[illegible] | NR2F1-AS1 | HSPA8P4 | LINC02057 |
| RN7SL2 | HLA-F-AS1 | LINC02984 | LINC01085 | LINC02212 |
| H4C5 | BASP1-AS1 | APOBEC3D | MTND5P9 | YWHAEP4 |
| COA1 | LOC442155 | APOBEC3C | WSPAR | LINC02269 |
| TTC31 | MIR381HG | LINC01550 | LINC01265 | LOC101929773 |
| SLC2A11 | BOLA3-DT | MGC16275 | LINC02742 | LOC124900211 |
| ZNF660 | PLCB1-IT1 | PCED1B-AS1 | OR7E99P | AKIRIN1P1 |
| ZNF135 | LRP8-DT | LINC02432 | LOC101060[illegible] | LOC124903387 |
| LINC00174 | PTPRD-AS1 | LOC442497 | RIPK2-DT | THRAP3P2 |
| ZNF662 | MIAT | PLBD1-AS1 | RNU6-1288[illegible] | LOC124903776 |
| ZNF793 | RPL7P3 | LCIIAR | RN7SKP12 | LOC101928708 |

| | | | | |
|---|---|---|---|---|
| ZNF789 | KRT16P6 | ARHGEF17-A | RN7SKP258 | CLEC19A |
| SRRM2-AS1 | LINC00348 | DIO3OS | RNU7-35P | LINC01613 |
| TRAJ46 | LINC01630 | LINC01833 | RNU6-299P | LINC02582 |
| TRAJ44 | CNTNAP5-DT | CEROX1 | LINC02159 | RN7SL823P |
| TRAJ43 | MIR34AHG | RNF157-AS1 | PCAT1 | MIR3193 |
| TRAJ42 | HDAC2-AS2 | LOC1249046 | MIR3142HG | LINC01892 |
| TRAJ41 | LINC01128 | IGF2-AS | NRG1-IT3 | ZSCAN5DP |
| TRAJ40 | LRRC3-DT | MXRA5 | MTCO1P47 | LOC100420254 |
| TRAJ39 | LINC01435 | ACTR3C | LINC03019 | MIR139 |
| TRAJ38 | LINC02723 | ABCC11 | C1DP5 | MIR8061 |
| TRAJ35 | LINC00574 | KCNK17 | TMEM123-DT | LOC124903525 |
| TRAJ33 | LRRC8C-DT | PRDM16-DT | LOC102723544 | |
| TRAJ32 | LINC02518 | P2RY8 | FAM133GP | |
| TRAJ29 | RAB6D | CYP27C1 | RPL31P57 | |
| TRAJ28 | LOC1001294 | TRABD2A | NOVA1-DT | |
| TRAJ27 | AMMECR1LP1 | OR52B6 | LINC00645 | |
| TRAJ26 | LINC02609 | LOC400499 | LINC00639 | |
| TRAJ25 | OBI1-AS1 | RNU6-223P | LOC101928988 | |
| TRAJ24 | DPP10-AS1 | RNA5SP350 | LOC101929124 | |
| TRAJ23 | PNMA6A | RNU6-824P | SULT1C2P2 | |
| TRAJ20 | LINC02889 | RNU6-899P | LOXL1-AS1 | |
| TRAJ18 | LINC01920 | RNU6-944P | MIR378G | |
| TRAJ17 | SCN1A-AS1 | RNU4-84P | MIR4802 | |
| TRAJ14 | MIR600HG | RNU4-67P | MIR548AV | |
| TRAJ13 | LRRC52-AS1 | RNU4-56P | MIR378E | |
| TRAJ12 | DCTN1-AS1 | RNU1-43P | RN7SL474P | |
| TRAJ11 | LINC01143 | LOC1249005 | MIR3142 | |
| TRAJ8 | LINC00398 | LINC01993 | MIR3978 | |
| TRAJ7 | LOC1027240 | C1RL-AS1 | MIR3690 | |
| TRAJ3 | LINC03014 | HBZP1 | RN7SL543P | |
| SEC14L1P1 | RN7SL164P | RNU6-1067P | MIR4435-2 | |
| AFG3L1P | RPL23AP49 | MIR548C | LOC105372068 | |
| THBS3-AS1 | RPL7P18 | MIR204 | LINC01837 | |
| LINC00852 | LOC1079853 | TRAV14DV4 | LOC105372145 | |
| RPL9P11 | A2M-AS1 | IGHV3-15 | BNIP3P23 | |
| ARHGEF9-IT | LINC02202 | RNU11-6P | PLCE1-AS1 | |
| KRT8P15 | NUDT16L2P | LOC1249001 | LOC105372316 | |
| ZNF736 | LOC1053793 | LAP3P2 | LETM1P1 | |
| GHRLOS | LINC02217 | LOC1001327 | LOC100420430 | |
| CECR3 | LOC283731 | ZNF90 | RN7SL840P | |
| LINC01843 | LINC01948 | ANXA2R-AS1 | OR5BL1P | |
| RNU6ATAC12 | HHIP-AS1 | RPL10AP3 | RBM22P6 | |
| RN7SKP105 | LOC285626 | MIR99AHG | RN7SL185P | |
| PSMA3-AS1 | PVT1 | RN7SKP154 | MIR6821 | |
| MIR3117 | LINC02891 | RNA5SP44 | MIR6731 | |
| ASB16-AS1 | TUNAR | RN7SKP194 | VNN2 | |
| ZNF790-AS1 | EPHA5-AS1 | RNU6-954P | H19 | |
| NBPF9 | AIDAP2 | LINC01342 | PLEKHG4B | |
| MIR6772 | ZFHX4-AS1 | LINC01857 | CPAMD8 | |
